# An inhibitory acetylcholine receptor gates context-dependent mechanosensory processing in *C. elegans*

**DOI:** 10.1016/j.isci.2024.110776

**Published:** 2024-08-22

**Authors:** Sandeep Kumar, Anuj K. Sharma, Andrew M. Leifer

**Affiliations:** 1 Princeton Neuroscience Institute, Princeton University, Princeton, NJ 08544, USA; 2 Department of Physics, Princeton University, Princeton, NJ 08544, USA

**Keywords:** Biological sciences, Genetics, Neuroscience

## Abstract

An animal’s current behavior influences its response to sensory stimuli, but the molecular and circuit-level mechanisms of this context-dependent decision-making are not well understood. *Caenorhabditis elegans* are less likely to respond to a mechanosensory stimulus by reversing if the stimuli is received while the animal turns. Inhibitory feedback from turning associated neurons are needed for this gating. But until now, it has remained unknown precisely where in the circuit gating occurs and which specific neurons and receptors receive inhibition from the turning circuitry. Here, we use genetic manipulations, single-cell rescue experiments, and high-throughput closed-loop optogenetic perturbations during behavior to reveal the specific neuron and receptor responsible for receiving inhibition and altering sensorimotor processing. Our measurements show that an inhibitory acetylcholine-gated chloride channel comprising LGC-47 and ACC-1 expressed in neuron type RIM disrupts mechanosensory evoked reversals during turns, presumably in response to inhibitory signals from turning-associated neuron SAA.

## Highlights

• Worms are less likely to reverse in response to touch stimuli delivered during turns
• Inhibitory acetylcholine receptor LGC-47 and ACC-1 in neuron RIM gates the response
• Presynaptic turning neuron SAA is required and is the presumed source of inhibition
• Therefore, feedback from SAA gates reversals by inhibiting RIM via LGC-47 and ACC-1

## Graphical abstract

**Figure:**
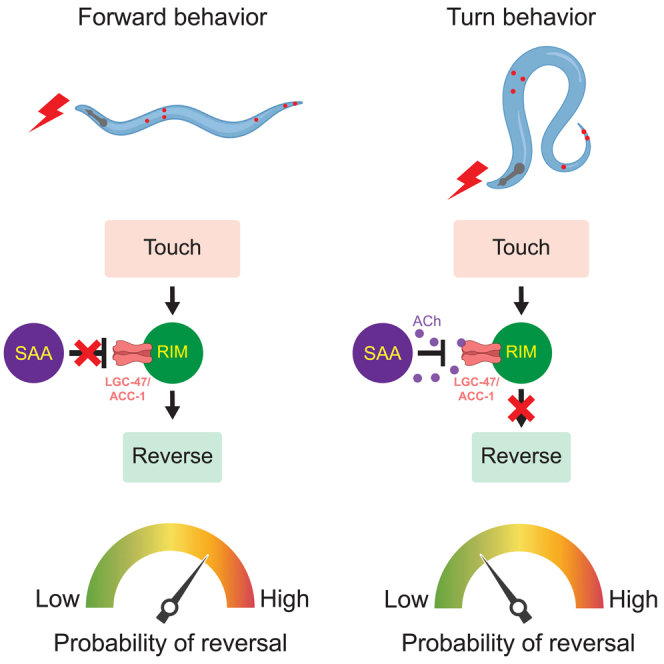

## Introduction

Animals use context to inform their response to a stimulus. Context comes from environmental cues,^1^ the animal’s internal state, such as hunger^2^ or arousal,^3^^,^^4^^,^^5^ or from the animal’s current behavior. Large scale neural population recording studies have found neural correlates of an animal’s pose or motor behavior across the brain,^6^^,^^7^^,^^8^ including in downstream sensory processing areas, suggesting that the brain may be incorporating relevant behavioral context information in the same brain areas where downstream sensory signals are also processed. We sought to use the nematode *Caenorhabditis elegans* with its compact and tractable nervous system to investigate the neurons, circuit, and receptors that underlie context-dependent sensorimotor processing and decision-making.

The *C. elegans* gentle-touch response circuit is well-suited for investigating how behavior context informs sensorimotor processing. Six touch receptor neurons detect gentle touch and send signals to downstream interneurons to evoke a motor response.^9^^,^^10^ When all six touch receptor neurons are stimulated with a plate tap or via optogenetics the animal typically responds by moving backward, called a reversal.^11^^,^^12^^,^^13^^,^^14^^,^^15^

**Figure 1:**
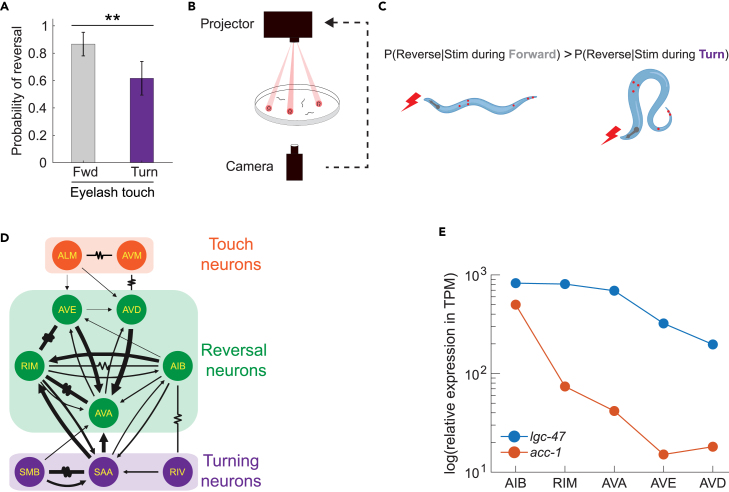
Mechanosensory evoked reversals are gated during turning via an unknown circuit- and molecular-mechanism (A) Mechanosensory stimuli is delivered to the anterior region of the worm using a classical manual eyelash touch assay. N=60 for both the forward and turn conditions. Data are represented as mean and 95% CI. p-value was determined via two proportion Z-test. ∗∗ indicates p<0.01. (B) Mechanosensory stimuli is delivered optogenetically to animals as they move using a custom targeted illumination system. (C) Illumination activates the optogenetic protein Chrimson expressed in the six gentle touch neurons. Stimulation delivered during forward movement is more likely to evoke reversals than stimuli delivered during the onset of a turn. Created with BioRender.com. (D) Inhibitory feedback from turning-associated neurons is hypothesized to gate the reversal response via an unknown circuit mechanism. Anatomical wiring diagram is shown of the anterior touch receptor neurons, reversal-associated interneurons and turning-associated neurons (adapted from Nemanode^21^). Arrows indicate chemical synapses. Resistor symbol indicates gap junctions. (E) Expression of two putative inhibitory acetylcholine receptor genes, *lgc-47* and *acc-1*, in selected interneurons. Relative expression is reported as RNA transcripts per million (TPM).^22^ Numerical values are listed in Table S1. All data underlying this figure is available at https://doi.org/10.6084/m9.figshare.25396453.

We previously discovered that *C. elegans*’ response to mechanosensory stimuli is influenced by its behavior context: it is less likely to reverse in response to a stimulus that it receives while executing a turn compared to a stimulus that it receives when moving forward (Figure 1C).^16^^,^^17^ This gating is visible in experiments with tap stimuli,^17^ optogenetic stimulation of mechanosensory neurons,^16^^,^^17^^,^^18^ and in a classical gentle-touch assay (Figure 1A). Suppressing a reversal response during turning may be ethologically beneficial because turns are a component of the *C. elegans*’ escape response,^19^^,^^20^ and ensuring that the turn completes may help preserve the animal’s ability to escape.^17^ Using optogenetics and a custom closed-loop high-throughput behavior assay as shown in Figure 1B, we previously found that activity from turning-associated neurons is needed to gate the mechanosensory evoked reversal response.^18^

In particular, activity from a collection of turning-associated neurons SMB, SAA, and RIV^23^ decreases the likelihood that a mechanosensory signal will interrupt a turn to evoke a reversal,^18^ presumably because these neurons send an inhibitory signal somewhere to the reversal circuitry. The exact location and mechanism with which this inhibitory feedback interacts with downstream mechanosensory processing is unknown. We previously surmised that inhibitory signals from turning neurons must arrive at or upstream of reversal interneuron AVA, because only activation of neurons upstream of AVA evoked reversals in a turning-dependent manner, while activation of AVA evoked reversals regardless of whether the animal had been turning or moving forward.^18^ Here, we seek to identify the precise neurons and receptors that receive inhibition from the turning circuit.

## Results

To find where in the network gating occurs, we sought to identify a reversal-associated neuron and receptor that receives inhibitory feedback from the turning-related neurons. We investigated post-synaptic partners of SAA because several strands of evidence pointed to SAA as a promising candidate for the source of turning-dependent inhibition: (1) SAA-type neurons are known to be involved in turning^23^^,^^24^^,^^25^^,^^26^; (2) SAA is one of three neuron subtypes that, when inhibited together, was sufficient to abolish turning-dependent gating^18^; and (3) SAA makes synaptic contacts onto key reversal interneurons including AVA, RIM, and AIB,^21^^,^^25^^,^^27^ Figure 1D.

We therefore searched for inhibitory acetylcholine receptors expressed postsynaptic of SAA. We looked for acetylcholine receptors because SAA is known to release acetylcholine^22^^,^^28^^,^^29^ and we sought those that were inhibitory because we expect SAA to send an inhibitory signal.^18^^,^^23^ We focused on *lgc-47* and *acc-1*, two genes for inhibitory acetylcholine receptors, with known expression in neurons RIM, AIB, and AVA among others, Table S1.^22^^,^^24^^,^^28^^,^^29^^,^^30^ RIM, AIB and AVA are of interest because they are well known to be involved in reversal behavior.^18^^,^^25^^,^^31^^,^^32^^,^^33^^,^^34^^,^^35^^,^^36^^,^^37^ We investigated LGC-47 first because it expresses at higher levels than ACC-1, Figure 1E.^22^

**Figure 2:**
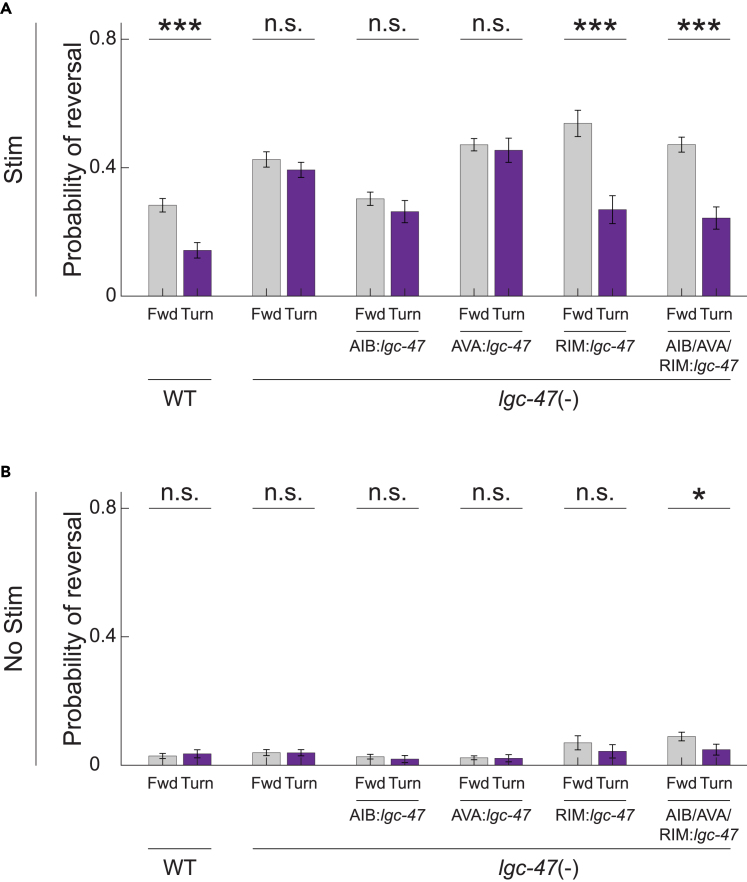
LGC-47 in neuron RIM gates turning-dependent reversals (A) Probability of reversal in response to optogenetic stimulation is shown for WT-background or *lgc-47* loss-of-function mutants. Cell specific rescues are performed to then restore *lgc-47* function only in specified neurons within the loss-of-function background. N>400 stimulation events per condition, reported in Table S2. p-value was determined via two proportion Z-test. ∗∗∗ indicates p<0.001/12, n.s. indicates p>0.05/12. Significance levels are chosen to account for a Bonferroni multiple hypothesis correction. All p-values are listed in Table S3. (B) Baseline reversal probabilities are shown for control experiments in which no optogenetic illumination is delivered. All animals express Chrimson under *mec-4* promoter including “WT.” N>369 mock stimulation events per condition, reported in Table S2. All p-values are listed in Table S3. Data are represented as mean and 95% CI. All data underlying this figure can be found at https://doi.org/10.6084/m9.figshare.25396453.

To investigate the role of LGC-47 in turning-dependent gating of mechanosensory evoked reversals, we measured the response to mechanosensory stimuli in *lgc-47* loss-of-function mutants. We expressed the light-gated ion channel Chrimson in the six gentle touch receptor neurons under the control of a *mec-4* promoter, and used a high throughput closed-loop optogenetic delivery system^16^ (Figure 1B) to automatically stimulate animals either when moving forward or triggered upon the onset of a turn, Figure 2. As expected, animals that received an 80 μW/${\text{mm}}^{2}$ intensity optogenetic light stimulus were more likely to respond by reversing (Figure 2A) than animals that received a 0 μW/${\text{mm}}^{2}$ “no-stim” control (Figure 2B).

Wild-type background animals gated their reversal response depending on whether they were turning, consistent with prior reports^16^^,^^17^^,^^18^: wild-type animals were significantly less likely to reverse in response to stimuli delivered while turning compared to stimuli delivered during forward movement, Figure 2. By contrast, *lgc-47* loss-of-function mutants exhibited little or no turning-dependent gating of the reversal response: they were similarly likely to reverse in response to stimuli regardless of whether the stimulus was delivered while the animal was turning or moving forward. Taken together, these measurements suggest that LGC-47 mediates gating of mechanosensory evoked reversals.

To identify where LGC-47 acts, we performed cell-specific rescues of LGC-47 function in the reversal associated interneurons AIB, AVA, RIM or all three, by re-expressing WT *lgc-47* cDNA in specific neurons within the *lgc-47* loss-of-function animals, Table 1. For each rescue, we measured the animal’s response to optogenetically induced mechanosensory stimuli, Figure 2. Only animals that expressed LGC-47 in RIM recapitulated the WT gating behavior. This shows that LGC-47 in RIM is necessary and sufficient to mediate gating. We therefore conclude that LGC-47 acts specifically in reversal neuron RIM to mediate the gating of mechanosensory evoked reversals.

**Table 1:** Genotype of strains used in this study

Strain name	Expression	Genotype	Background	Figure	Reference
N2	N/A	WT	WT	Figure 1	Caenorhabditis Genetics Center
AML67	Chrimson in ALML/R, AVM, PLML/R, PVM	*wtfIs46[Pmec-4::Chrimson::SL2::mCherry::unc- 54 40ng/ul]*	N2-WT	Figure 2	Liu et al.^17^
AML597	Chrimson in ALML/R, AVM, PLML/R, PVM	*lgc-47(sy1501) X; wtfIs46[mec-4P::Chrimson::SL2::mCherry::unc-54 40 ng/ul]*	*lgc-47 (sy1501)*	Figure 2	This work
AML617	Chrimson in ALML/R, AVM, PLML/R, PVM; rescuing lgc-47 in AIB neuron	*lgc-47(sy1501) X; wtfIs46[mec- 4P::Chrimson::SL2::mCherry::unc-54 40 ng/ul]; wtfEX538 [npr-9P::AI::lgc-47::SL2::tagBFP 30ng/ul + Coel::GFP 70ng/ul]*	*lgc-47 (sy1501)*	Figure 2	This work
AML618	Chrimson in ALML/R, AVM, PLML/R, PVM; rescuing lgc-47 in AVA neuron	*lgc-47(sy1501) X; wtfIs46[mec- 4P::Chrimson::SL2::mCherry::unc-54 40 ng/ul]; wtfEX539 [rig-3P::AI::lgc-47::SL2::GFP 30ng/ul + Coel::GFP 70ng/ul]*	*lgc-47 (sy1501)*	Figure 2	This work
AML614	Chrimson in ALML/R, AVM, PLML/R, PVM; rescuing lgc-47 in RIM neuron	*lgc-47(sy1501) X; wtfIs46[mec- 4P::Chrimson::SL2::mCherry::unc-54 40 ng/ul]; wtfEX535 [tdc-1P::AI::lgc-47::SL2::his- 24::tagRFP 30ng/ul + Coel::GFP 70ng/ul]*	*lgc-47 (sy1501)*	Figure 2	This work
AML622	Chrimson in ALML/R, AVM, PLML/R, PVM; rescuing lgc-47 in AIB, AVA, and RIM neuron	*lgc-47(sy1501) X; wtfIs46[mec- 4P::Chrimson::SL2::mCherry::unc-54 40 ng/ul]; wtfEX543 [tdc-1P::AI::lgc-47::SL2::his- 24::tagRFP 30ng/ul +npr-9P::AI::lgc- 47::SL2::tagBFP 30ng/ul + rig-3P::AI::lgc- 47::SL2::GFP 30ng/ul + Coel::GFP 70ng/ul]*	*lgc-47 (sy1501)*	Figure 2	This work
AML627	Chrimson in ALML/R, AVM, PLML/R, PVM	*acc-1 (tm3268)IV; wtfIs46[mec- 4P::Chrimson::SL2::mCherry::unc-54 40 ng/ul]*	*acc-1 (tm3268)*	Figure 3	This work
AML659	Chrimson in ALML/R, AVM, PLML/R, PVM	*acc-1 (tm3268)IV; lgc- 47(sy1501) X; wtfIs46[mec- 4P::Chrimson::SL2::mCherry::unc-54 40 ng/ul]*	*lgc-47 (sy1501)* and *acc-1 (tm3268)*	Figure 3	This work
WEN1015	Chrimson in ALML/R, AVM, PLML/R, PVM	*wenIs1015[Pmec-4::chrimson::mcherry(quan0047,50ng/ul), Plin-44::gfp]*	N2-WT	Figure 3	Huo et al.^24^
WEN1025	Chrimson in ALML/R, AVM, PLML/R, PVM	*acc-1(tm3268); wenIs1015[Pmec-4::Chrimson::mCherry; Plin-44::GFP]*	*acc-1 (tm3268)*	Figure 3	Huo et al.^24^
WEN0920	Chrimson in ALML/R, AVM, PLML/R, PVM; rescuing acc-1 in RIM neuron	*acc-1(tm3268); wenIs1015[Pmec-4::Chrimson::mCherry; Plin-44.:GFP]; wenEx0920[Ptdc1::acc1::GFP(20ng/ul); Plin-44::mcherry]*	*acc-1 (tm3268)*	Figure 3	Huo et al.^24^

We next investigated the candidate inhibitory acetylcholine receptor gene *acc-1* that is expressed in a similar pattern of neurons, albeit at a lower expression level Figure 1E.^22^^,^^28^^,^^30^ Intriguingly, a recent study suggests that ACC-1 regulates the duration of spontaneous reversals in a manner similar to what we hypothesize for LGC-47: namely ACC-1 is thought to inhibit RIM upon SAA release of acetylcholine, in that case halting an ongoing reversal.^24^ We wondered whether, in addition to LGC-47, ACC-1 also contributes to turning-dependent gating by potentially stopping reversals before they start.

**Figure 3:**
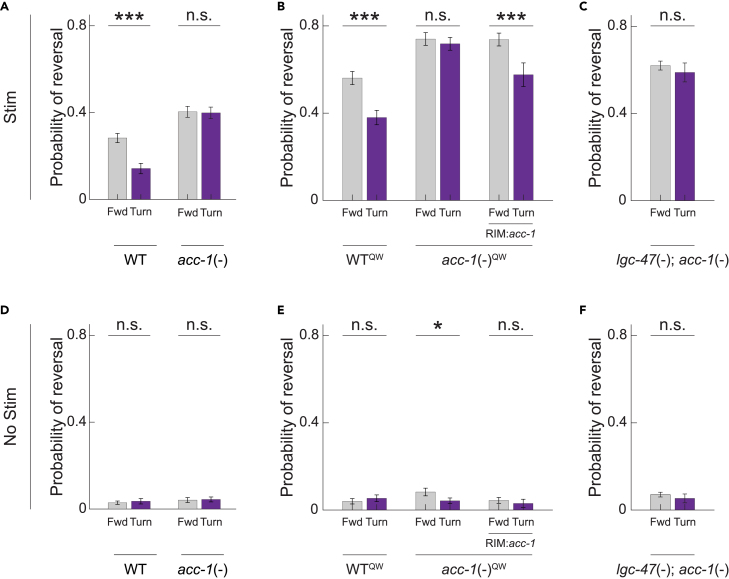
ACC-1 in neuron RIM also gates turning-dependent reversals (A) Probability of reversal in response to optogenetic stimulation delivered during forward movement or turning onset is shown for “WT” and *acc-1* loss-of-function mutants using the same P*mec-4::Chrimson* strain as in Figure 2. For all panels in this figure, p-value was determined via two proportion Z-test. ∗∗∗ indicates p<0.001/12, n.s. indicates p>0.05/12. (B) Restoring *acc-1* function only in neuron RIM rescues the gating. Here a different *mec-4* Chrimson allele is used, denoted by QW. (C) Probability of reversal for a double mutant genetic strain with *lgc-47* and *acc-1* loss-of-function mutants using the same P*mec-4::Chrimson* strain as in panel A). N>320 stimulation events per condition. (D–F) Baseline reversal probabilities are shown for control experiments in which no optogenetic illumination is delivered. N>298 mock stimulation events per condition. Exact number of stimulus events per condition are listed in Table S2. Significance levels are chosen to account for a Bonferroni multiple hypothesis correction. p-values for all the comparisons are listed in Table S3. Data are represented as mean and 95% CI. All data underlying this figure is available at https://doi.org/10.6084/m9.figshare.25396453.

*acc-1* loss-of-function mutants showed no turning-dependence in their mechanosensory evoked responses (Figure 3) just like *lgc-47* mutants. We observed the same effect both in our *mec-4::Chrimson* background strains (Figure 3A) and in a nominally similar but separately generated set of strains from the Quan Wen group, Figure 3B. Turning dependence reappeared when ACC-1 was rescued in the neuron type RIM, just as it did for LGC-47. Therefore ACC-1 in RIM performs the same role as LGC-47 in RIM– both mediate turning-dependent gating.

Recent *in vitro* electrophysiology studies suggest that LGC-47 and ACC-1 may form a heteromeric ion channel.^38^^,^^39^ and our finding that LGC-47 and ACC-1 perform the same role *in vivo* supports this hypothesis. We therefore measured responses to mechanosensory stimuli in *acc-1*/*lgc-47* loss-of-function double mutants that lacked functional copies of both LGC-47 and ACC-1. Worms in this double mutant background also showed little or no gating, Figure 3C. This further supports our claims that these inhibitory acetylcholine receptors mediate the turning-dependent gating of reversals by inhibiting RIM.

What might be the source of the inhibitory acetylcholine signal that acts on LGC-47 and ACC-1 to inhibit RIM? Several lines of evidence point strongly to SAA as the source of the inhibitory acetylcholine signal. SAA is notably one of the few neurons that serve the correct functional role: We previously observed that inhibiting SAA, RIV, and SMB together is sufficient to stop the gating^18^ indicating that at least one of those neurons is needed for gating. Of those three, only SAA makes synaptic contacts onto RIM^21^^,^^27^ and is therefore the only one that is wired correctly to directly inhibit RIM. Moreover, the SAA to RIM connection has a large “synapse count” suggesting a strong connection from SAA to RIM. Importantly, SAA releases acetylcholine,^28^^,^^29^ the appropriate transmitter to inhibit RIM via LGC-47 and ACC-1. SAA is also active at the right time– calcium imaging experiments have shown that SAA is active during the onset of turning,^24^ which is when we expect an acetylcholine signal to arrive. Finally, optogenetics and calcium imaging experiments in Ref.^24^ show that SAA inhibits RIM, albeit in a different context. This suggests that SAA could also inhibit RIM during turning to gate mechanosensory evoked reversals. All of this evidence strongly suggests that SAA is likely the source of acetylcholine that inhibits RIM via LGC-47 and ACC-1.

## Discussion and conclusions

**Figure 4:**
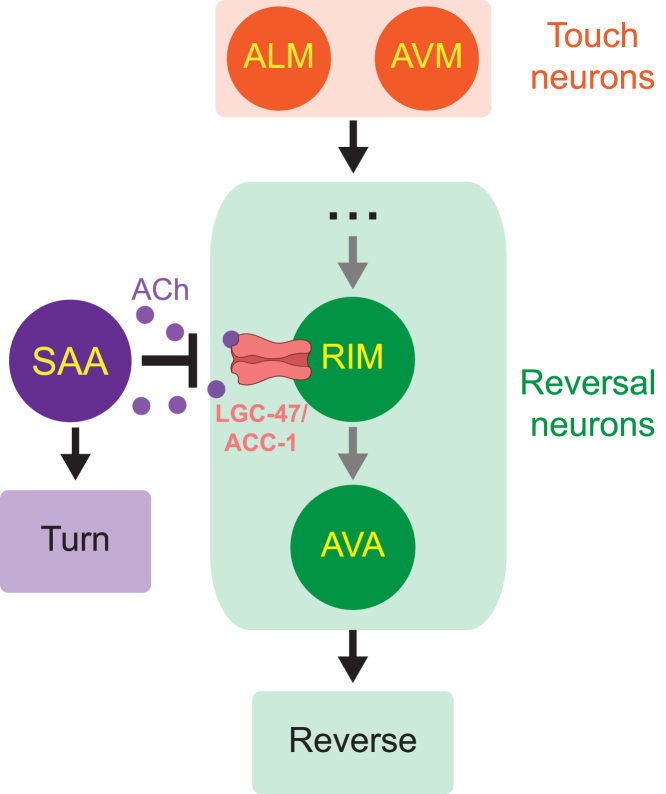
Proposed mechanism for turning-dependent gating of the reversal response SAA is active during turns and releases acetylcholine that binds LGC-47/ACC-1 and inhibits RIM. Inhibition of RIM (and, by extension, its gap-junction partners) disrupts mechanosensory signals originating from the touch neurons and prevents them from traveling downstream to activate reversal command neuron AVA. As a result, the probability that a mechanosensory stimulus evokes a reversal decreases. Here arrows represent information flow, not necessarily synaptic transmission.

Taken together, we conclude that turning gates mechanosensory evoked reversals by inhibiting reversal neurons RIM via an inhibitory acetylcholine receptor comprising LGC-47 and ACC-1, Figure 4. Strong evidence from others in the literature^24^ and from our prior work^18^ further suggests that SAA is the source of the inhibitory acetylcholine signal that acts on LGC-47 and ACC-1. This work is consistent with and adds critical missing mechanistic details to our prior findings that turning-related signals arrive somewhere at or upstream of neuron AVA to prevent reversals.^18^ Inhibition of RIM during turns is ideally situated to prevent reversals because it makes many gap junctions with neurons AVA and AVE, as well as gap junctions with AIB--- all neurons implicated in promoting reversals. We previously found that once AVA is active, reversals are no longer dependent on turning,^18^ and that activity in AVA more closely reflects behavioral output rather than sensory input.^40^ Inhibition of RIM therefore may serve as a shunt to inhibit activity across the reversal circuitry and prevent AVA from becoming active. Indeed chronic inhibition of RIM is known to suppress reversals in other contexts.^37^ Future functional imaging studies are needed to reveal the neural dynamics of these neurons in response to mechanosensory stimuli.

We investigated the role of LGC-47 and ACC-1, and concluded that they are responsible for receiving inhibitory feedback at RIM and performing the gating. Our findings are consistent with the hypothesis that these two proteins form a heteromeric inhibitory ion channel.^38^^,^^39^ In addition, it is possible that other proteins including ACC-2, ACC-3 and ACC-4 may also contribute as subunits or heteromers of this inhibitory receptor.^24^^,^^30^ Future work is needed to explore their roles.

Context-dependent sensory processing is a common feature of the nervous system, even beyond the worm. In the mammalian literature, there is extensive evidence that sensory perceptions are modulated by the context in which they are experienced.^41^ For example, locomotion is known to modulate neural responses to visual stimuli, likely by a disinhibition circuit.^6^^,^^42^ A challenge with studying circuit level mechanisms of context-dependent processing in mammalian systems is that mammal’s large brains can make it difficult to trace neural signals from motor to sensory areas. We are able to investigate molecular and circuit mechanisms more comprehensively by working in the small nematode *C. elegans*.

We have shown how the worm nervous system implements a context-dependent decision to reverse in response to stimuli. Our proposed circuit spans from motor to sensory and back, Figure 4. Specifically, inhibitory feedback from motor-related turning neurons are combined with downstream mechanosensory signals at a single interneuron pair by a single ion receptor type to gate the animal’s motor response to stimuli. The convergence at a single receptor type on a single neuron pair is striking, and may reflect the unique constraints imposed by the worm’s small nervous system of only 302 neurons. Even so, the broad approach of combining inhibitory motor feedback with sensory signals to modulate a sensorimotor response is accessible to many organisms and may therefore be a general feature of context-dependent decision-making.

### Limitations of the study

This study investigates only two inhibitory acetylcholine receptors, LGC-47 and ACC-1. Future work is needed to investigate other potential contributors including ACC-2, ACC-3, and ACC-4.^24^ In this work we relied on our prior findings^18^ and on strong additional evidence from the literature^24^ to conclude that presynaptic neuron SAA is the relevant source of inhibition onto RIM. Future cell-specific manipulations of SAA could provide further evidence to support this conclusion. Finally, this work leverages the power of high-throughput optogenetics, automated computer vision, and cell-resolved genetic manipulations to dissect the neural circuit and molecular mechanisms underlying a sensorimotor process. An alternative approach would be to use optical physiology to measure neural dynamics during behavior directly.^43^^,^^44^

## Resource availability

### Lead contact

Further information and requests for resources and reagents should be directed to and will be fulfilled by the lead contact, A.L. (leifer@princeton.edu).

### Materials availability

All the plasmids and transgenic strains AML67, AML597, AML614, AML617, AML618, AML622, AML627, and AML659 are being made publicly available through Addgene and the Caenorhabditis Genetics Center (CGC), respectively.

### Data and code availability

• Computer-readable files showing processed tracked behavior and optogenetic stimulus events for all experiments are publicly accessible at https://doi.org/10.6084/m9.figshare.25396453.
• All analysis codes used in this manuscript are publicly accessible at https://github.com/leiferlab/kumar-molecular-mechanism.git.

## Acknowledgments

We thank Dr. Quan Wen (University of Science and Technology of China) and Dr. Cori Bargmann for strains. This work used computing resources from the Princeton Institute for Computational Science and Engineering. Strains newly generated by this work are distributed by the CGC, which is funded by the 10.13039/100000002NIH 10.13039/100016958Office of Research Infrastructure Programs (P40 OD010440). The research reported in this work was supported by the 10.13039/100000001National Science Foundation (https://www.nsf.gov) through an NSF CAREER Award to A.M.L. (IOS-1845137) and through the Center for the Physics of Biological Function (PHY-1734030); and by the 10.13039/100000065National Institute of Neurological Disorders and Stroke (https://www.ninds.nih.gov/) of the 10.13039/100000002National Institutes of Health, 10.13039/100000065National Institute of Neurological Disorders and Stroke under New Innovator award number DP2-NS116768 to A.M.L.; and by the 10.13039/100000893Simons Foundation (https://www.simonsfoundation.org/) under award SCGB 543003 to A.M.L. The funders had no role in study design, data collection and analysis, decision to publish, or preparation of the manuscript. We acknowledge BioRender.com for use of graphics.

## Author contributions

Conceptualization: A.M.L. and S.K.; Formal analysis: S.K.; Funding acquisition: A.M.L.; Investigation: S.K.; Methodology: S.K. and A.K.S.; Project administration: A.M.L.; Resources: A.K.S.; Supervision: A.M.L.; Writing – original draft: S.K.; Writing – review and editing: A.M.L., S.K., and A.K.S.

## Declaration of interests

The authors declare no competing interests.

## STAR★Methods

### Key resources table

**Table:** 

REAGENT or RESOURCE	SOURCE	IDENTIFIER
**Bacterial and virus strains**		

*E. coli* (OP50)	Caenorhabditis Genetics Center	WBStrain00041969
*E. coli* (HST08), Stellar Competent Cells	Takara	Cat. # 636763

**Chemicals, peptides, and recombinant proteins**		

All Trans Retinal (ATR)	Sigma-Aldrich	Cat. # R2500
KH_2_PO_4_ (for M9)	Fisher Chemicals	Cat. # P285
Na_2_HPO_4_ (for M9)	Thermo Scientific	Cat. # 013437.A1
NaCl (for M9)	Fisher Chemicals	Cat. # S271
MgSO_4_ (Anhydrous) (for M9)	Fisher Chemicals	Cat. # M65
Sodium Hypochlorite 12.5% (Bleach)	VWR Chemicals	Cat. # BDH7038
NaOH	Fisher Scientific	Cat. # BP359
Agarose	Calbiochem	Cat. # 2125
Ethidium Bromide (EtBr)	Sigma-Aldrich	Cat. # E1510

**Critical commercial assays**		

In-Fusion Snap Assembly Master Mix	Takara	Cat. # 638948
PrimeSTAR GXL DNA Polymerase	Takara	Cat. # R050
NucleoSpin Gel and PCR Clean-Up	Takara	Cat. # 740609
PureLink Quick Plasmid Miniprep Kit	Invitrogen	Cat. # K210011

**Deposited data**		

Original code and data related to recording and analyzing behavior	This paper	https://doi.org/10.6084/m9.figshare.25396453

**Experimental models: Organisms/strains**		

N2	Caenorhabditis Genetics Center	WBStrain00000001
*C. elegans*: wtfIs46[Pmec-4::Chrimson::SL2::mCherry::unc-54 40ng/ul]	Liu et al.^17^	AML67, WBStrain00000193
*C. elegans*: lgc-47(sy1501) X; wtfIs46[mec-4P::Chrimson::SL2::mCherry::unc-54 40ng/ul]	This paper	AML597
*C. elegans*: lgc-47(sy1501) X; wtfIs46[mec-4P::Chrimson::SL2::mCherry::unc-54 40ng/ul]; wtfEX535 [tdc-1P::AI::lgc-47::SL2::his-24::tagRFP 30ng/ul + Coel::GFP 70ng/ul]	This paper	AML614
*C. elegans*: lgc-47(sy1501) X; wtfIs46[mec-4P::Chrimson::SL2::mCherry::unc-54 40 ng/ul]; wtfEX538 [npr-9P::AI::lgc-47::SL2::tagBFP30ng/ul + Coel::GFP 70ng/ul]	This paper	AML617
*C. elegans*: lgc-47(sy1501) X; wtfIs46[mec-4P::Chrimson::SL2::mCherry::unc-54 40ng/ul]; wtfEX539 [rig-3P::AI::lgc-47::SL2::GFP30ng/ul + Coel::GFP 70ng/ul]	This paper	AML618
*C. elegans*: lgc-47(sy1501) X; wtfIs46[mec-4P::Chrimson::SL2::mCherry::unc-54 40ng/ul]; wtfEX543 [tdc-1P::AI::lgc-47::SL2::his-24::tagRFP 30ng/ul +npr-9P::AI::lgc-47::SL2::tagBFP 30ng/ul + rig-3P::AI::lgc-47::SL2::GFP 30ng/ul + Coel::GFP 70ng/ul]	This paper	AML622
*C. elegans*: acc-1 (tm3268)IV; wtfIs46[mec-4P::Chrimson::SL2::mCherry::unc-54 40ng/ul]	This paper	AML627
*C. elegans*: acc-1 (tm3268)IV; lgc- 47(sy1501) X; wtfIs46[mec-4P::Chrimson::SL2::mCherry::unc-54 40 ng/ul]	This paper	AML659
*C. elegans*: wenIs1015[Pmec-4::chrimson::mcherry(quan0047,50ng/ul), Plin-44::gfp]	Huo et al.^24^	WEN1015
*C. elegans*: acc-1(tm3268); wenIs1015[Pmec-4::Chrimson::mCherry; Plin-44::GFP]	Huo et al.^24^	WEN1025
*C. elegans*: acc-1(tm3268); wenIs1015[Pmec-4::Chrimson::mCherry; Plin-44.:GFP]; wenEx0920[Ptdc1::acc1::GFP(20ng/ul); Plin-44::mcherry]	Huo et al.^24^	WEN0920

**Recombinant DNA**		

Plasmid: mec-4P::Chrimson::SL2::mCherry::unc-54-3’UTR	Liu et al.^17^	Addgene: Plasmid #107745
Plasmid: npr-9P::AI::LGC-47::SL2::tagBFP::unc-54-3′UTR	This paper	RRID: Addgene_225923
Plasmid: rig-3P::AI::LGC-47::SL2::GFP::unc-54-3′UTR	This paper	RRID: Addgene_225924
Plasmid: tdc-1P::AI::LGC-47::SL2::his-24::tagRFP::unc-54-3′UTR	This paper	RRID: Addgene_225925

**Software and algorithms**		

Analysis code	This paper	https://github.com/leiferlab/kumar-molecular-mechanism.git
MATLAB	MathWorks	https://www.mathworks.com/products/matlab.html
LabVIEW	National Instruments	https://www.ni.com/en/shop/labview.html
BioRender	BioRender	https://biorender.com/
Adobe Illustrator	Adobe	https://www.adobe.com

**Other**		

Unseeded NGM plate, 100 × 15mm (for behavior assay)	LabExpress	Cat. # 5001-100

### Experimental model and study participant details

#### Nematode handling

Worm handling was performed as described in Ref.^18^ Briefly, all the strains used in this study were grown on standard nematode growth media plates with OP-50 (*E. coli*) as a food source at 20 C. Agarose plates containing gravid worms were bleached to collect eggs. The eggs were rinsed with M9 solution at least three times and left on a shaker overnight. The next day, L1 larvae were plated on a freshly seeded plate containing OP50 mixed with 1 mL of 0.5 mM all-trans-retinal and placed in a dark container in a 20 C incubator until day 1 young adult stage, at which time experiments were performed.

#### *C. elegans* strains

All strains used in this work are listed in Table 1. The CRISPR engineered null mutant strain PS8742 [*lgc-47(sy1501)*] for the LGC-47 receptor was obtained from the Caenorhabditis Genetics Center (CGC). The mutant strain CX12721 [*acc-1(tm3268)*] defective for the ACC-1 receptor was a gift from Dr. Cori Bargmann, Rockefeller University. To rescue the *lgc-47* cDNA in neurons RIM, AIB, and AVA, we used the cell specific promoters *tdc-1P*, *npr-9P*, and *rig-3P* respectively. Strains WEN0920, WEN1015, and WEN1025, were gifts from Dr. Quan Wen, University of Science and Technology of China. RNA expression levels of *lgc-47* and *acc-1* were reported from the CeNGEN database^22^ and displayed in Table S1.

### Method details

#### Optogenetic stimulation

To measure the response of mechanosensory stimulus during forward and turning behaviors, we delivered optogenetic stimulus to the worms using a high throughput optogenetic delivery system.^16^ Optogenetic stimulation was performed as described previously.^18^ Briefly, an open-loop optogenetic stimulation protocol was used to stimulate the animal when the animal was moving forward. 3 s of 80 μ W/${\text{mm}}^{2}$, 630 nm illumination was delivered to all animals on the plate every 30 s. Only stimuli that landed when the animal was moving forward were considered. To investigate the behavioral response to stimuli during turns, a closed-loop behavior-triggered stimulus was used. 3 s of illumination was delivered to an animal whenever the system detected that a worm was initiating a turn, but no more often than once every 30 s to the same animal.

#### Eyelash touch assay

The protocol used to deliver touch stimulus to the worm using an eyelash is described in detail in a previous work.^45^ Worms were transferred to a plain agarose plate and allowed to roam freely for at least 2 min to let them acclimate to the new plate. An eyelash was sterilized using 70% ethanol. It was then used to deliver a gentle touch stimulus to the anterior region of the worm’s body when the worm was either moving forward or making a turn. Reversal responses were scored manually. Only one stimulus was delivered to each of the worms.

#### Behavior analysis

Behavior classification of forward movements, turns and reversals was performed as described previously.^18^ Briefly, two sets of behavior mapping algorithms were used in this study, one for real-time tracking of worms and optogenetic stimulation, and another more conservative one for post-processing analysis. The real-time algorithm tracked each worm as it was crawling on agarose plates and determined locomotory parameters in real-time such as velocity, centerline, body curvature, etc. That algorithm is used for behavior-triggered stimulation. E.g., when the system detects that the worm is initiating a turn, a computer controlled projector delivers an optogenetic stimulus precisely to that worm. In post-processing, we classify the worm’s behavior, and determine whether the worm reversed in response to stimuli.^17^^,^^46^ We exclude worms that do not move for prolonged periods of time. A worm is classified as reversing in response to stimuli if its velocity is less than or equal to −0.11 mm/s during the 3 s optogenetic stimulation window. The number of experimental assays and stimulus events can be found in Table S2.

### Quantification and statistical analysis

In our analysis, stimulus events are the fundamental units, and we calculate statistics based on the probability of exhibiting a response to a stimulus event. We report three statistics: the proportion of stimulus events that resulted in a reversal of the worm, the total number of stimulus events presented to the worm, and the corresponding 95% confidence interval for the proportions of the worm reversing. To reject the null hypothesis that two empirically observed proportions (for example during forward and turning) are the same, we used a two-proportion Z-test and reported a p-value.^47^ A p-value < 0.05 after Bonferroni correction was considered significant.
