# Supplementary material for: An inhibitory acetylcholine receptor gates context-dependent mechanosensory processing in C. elegans
Source: iScience. 2024 Aug 22;27(10):110776. doi: 10.1016/j.isci.2024.110776 (PMC11460506; doi:10.1016/j.isci.2024.110776)
Supplement: Document S1. Tables S1–S3 [file mmc1.pdf]

iScience, Volume *27*

Supplemental information

An inhibitory acetylcholine receptor gates context-dependent mechanosensory processing in *C. elegans*

Sandeep Kumar, Anuj K. Sharma, and Andrew M. Leifer

	Neuron	*lgc-47* (in TPM)	*acc-1* (in TPM)
Sensory neurons	ALM	24.8	20
	AVM	9.7	7.8
Reversal interneurons	AVD	197.2	18.1
	AVE	322.1	15.1
	AVA	691.6	41.7
	RIM	808.1	73.9
	AIB	825.2	499.5
Turning neurons	RIV	520.6	5.74
	SMB	558.6	30.1
	SAA	1417.6	497.7

Table S1. Reported receptor expression levels, related to Figure 1E.

Relative expression levels of lgc-47 and acc-1 in transcripts per million (TPM) as reported in CeNGEN single cell gene expression atlas[S1] (no threshold was selected).

Strain name	Target Behavior	# Assays	Illumination Intensity ($\mu W/mm^2$)	# stim events	Figures
N2	Forward	N/A	N/A (eyelash touch)	60	Fig 1
N2	Turn	N/A	N/A (eyelash touch)	60	Fig 1
AML67	Forward	4	80	1,766	Fig 2, Fig 3
AML67	Forward	4	0	1,729	Fig 2, Fig 3
AML67	Turn	9	80	806	Fig 2, Fig 3
AML67	Turn	9	0	842	Fig 2, Fig 3
AML597	Forward	3	80	1,652	Fig 2
AML597	Forward	3	0	1,612	Fig 2
AML597	Turn	15	80	1,643	Fig 2
AML597	Turn	15	0	1,548	Fig 2
AML617	Forward	11	80	1,890	Fig 2
AML617	Forward	11	0	1,842	Fig 2
AML617	Turn	22	80	623	Fig 2
AML617	Turn	22	0	575	Fig 2
AML618	Forward	5	80	2,598	Fig 2
AML618	Forward	5	0	2,382	Fig 2
AML618	Turn	12	80	667	Fig 2
AML618	Turn	12	0	648	Fig 2
AML614	Forward	3	80	567	Fig 2
AML614	Forward	3	0	516	Fig 2
AML614	Turn	24	80	401	Fig 2
AML614	Turn	24	0	370	Fig 2
AML622	Forward	8	80	1,755	Fig 2
AML622	Forward	8	0	1,721	Fig 2
AML622	Turn	15	80	592	Fig 2
AML622	Turn	15	0	619	Fig 2
AML627	Forward	3	80	1,424	Fig 3
AML627	Forward	3	0	1,188	Fig 3
AML627	Turn	16	80	1,301	Fig 3
AML627	Turn	16	0	1,271	Fig 3
AML659	Forward	3	80	2,142	Fig 3
AML659	Forward	3	0	2,160	Fig 3
AML659	Turn	3	80	493	Fig 3
AML659	Turn	3	0	469	Fig 3
WEN1015	Forward	3	80	1,048	Fig 3
WEN1015	Forward	3	0	973	Fig 3
WEN1015	Turn	15	80	821	Fig 3
WEN1015	Turn	15	0	817	Fig 3
WEN1025	Forward	4	80	869	Fig 3
WEN1025	Forward	4	0	932	Fig 3
WEN1025	Turn	14	80	904	Fig 3
WEN1025	Turn	14	0	945	Fig 3
WEN0920	Forward	4	80	853	Fig 3
WEN0920	Forward	4	0	906	Fig 3
WEN0920	Turn	21	80	321	Fig 3
WEN0920	Turn	21	0	299	Fig 3

Table S2. Details of experiments performed, related to Figure 1, Figure 2, and Figure 3. Strains used, number of assays, optogenetic illumination intensity, and number of stimulation events are listed for each experiment.

Figure 1A			
Condition	Condition	p-value	Significance after Bonferroni correction
N2 (Fwd)	N2 (Turn)	0.0022	**

Figure 2A			
Condition	Condition	p-value	Significance after Bonferroni correction
AML67 (Fwd)	AML67 (Turn)	1.35E-14	***
AML597 (Fwd)	AML597 (Turn)	0.0589	n.s.
AML617 (Fwd)	AML617 (Turn)	0.0577	n.s.
AML618 (Fwd)	AML618 (Turn)	0.426	n.s.
AML614 (Fwd)	AML614(Turn)	2.22E-16	***
AML622 (Fwd)	AML622 (Turn)	<E-324	***

Figure 2B			
Condition	Condition	p-value	Significance after Bonferroni correction
AML67 (Fwd)	AML67 (Turn)	0.3578	n.s.
AML597 (Fwd)	AML597 (Turn)	0.9627	n.s.
AML617 (Fwd)	AML617 (Turn)	0.3149	n.s.
AML618 (Fwd)	AML618 (Turn)	0.8223	n.s.
AML614 (Fwd)	AML614(Turn)	0.098	n.s.
AML622 (Fwd)	AML622 (Turn)	0.0012	*

Figure 3A			
Condition	Condition	p-value	Significance after Bonferroni correction
AML67 (Fwd)	AML67 (Turn)	1.35E-14	***
AML627 (Fwd)	AML627 (Turn)	0.7957	n.s.

Figure 3B			
Condition	Condition	p-value	Significance after Bonferroni correction
WEN1015 (Fwd)	WEN1015 (Turn)	1.24E-14	***
WEN1025 (Fwd)	WEN1025 (Turn)	0.29754	n.s.
WEN0920 (Fwd)	WEN0920 (Turn)	1.15E-07	***

Figure 3C			
Condition	Condition	p-value	Significance after Bonferroni correction
AML659 (Fwd)	AML659 (Turn)	0.1919	n.s.

Figure 3D			
Condition	Condition	p-value	Significance after Bonferroni correction
AML67 (Fwd)	AML67 (Turn)	0.3578	n.s.
AML627 (Fwd)	AML627 (Turn)	0.7302	n.s.

Figure 3E			
Condition	Condition	p-value	Significance after Bonferroni correction
WEN1015 (Fwd)	WEN1015 (Turn)	0.16766	n.s.
WEN1025 (Fwd)	WEN1025 (Turn)	3.14E-04	*
WEN0920 (Fwd)	WEN0920 (Turn)	0.28638	n.s.

Figure 3F			
Condition	Condition	p-value	Significance after Bonferroni correction
AML659 (Fwd)	AML659 (Turn)	0.1710	n.s.

Table S3. The p-values corresponding to all comparisons reported in all figures in this manuscript, related to Figure 1, Figure 2, and Figure 3. The p-values are calculated via a two-proportion Z-test. Statistical significance was determined after Bonferroni correction.

Supplementary References:

S1 Taylor, S. R., Santpere, G., Weinreb, A., Barrett, A., Reilly, M. B., Xu, C., Varol, E., Oikonomou, P., Glenwinkel, L., McWhirter, R., et al. (2021). Molecular topography of an entire nervous system. en. Cell 184, 4329–4347.e23. https://doi.org/10.1016/j.cell.2021.06.023.
